# Detection of Crimean-Congo hemorrhagic fever virus and Rift Valley fever virus antibodies in animal workers in Cameroon

**DOI:** 10.3389/fvets.2025.1646715

**Published:** 2025-11-28

**Authors:** Gisele Liliane Machuetum, Jules Brice Tchatchueng-Mbougua, Yannick Munyeku-Bazitama, Keita Mizuma, Gael Essima, Landry Mounchili, Christian Yogne Nsangou, Jean Dominique Mbarga Owona, Delia Doreen Djuicy, Martial Yonga, Maloum Souleymanou, Rodrigue Poueme, Basile Kamgang, Ahmadou Alkaissou, Alain Bertrand Dongmo, Francioli Koro Koro, Ayato Takada, Keita Matsuno, Paul Alain Tagnouokam-Ngoupo, Richard Njouom

**Affiliations:** 1 Centre Pasteur du Cameroun, Yaoundé, Cameroon; 2 University of Douala, Douala, Cameroon; 3 International Institute for Zoonosis Control, Hokkaido University, Sapporo, Japan; 4 Institut National de Recherche Biomédicale, Kinshasa, Democratic Republic of Congo; 5 Laboratoire National Vétérinaire, Garoua, Cameroon; 6 Centre for Research in Infectious Diseases, Yaoundé, Cameroon; 7 Ministry of Livestock, Fisheries and Animal Industries, Yaoundé, Cameroon; 8 One Health Research Center, Hokkaido University, Sapporo, Japan; 9 School of Veterinary Medicine, University of Zambia, Lusaka, Zambia; 10 Institute for Vaccine Research and Development (HU-IVReD), Hokkaido University, Sapporo, Japan

**Keywords:** Rift Valley fever, Crimean-Congo hemorrhagic fever, seroprevalence, associated factors, animal workers, Cameroon

## Abstract

**Introduction:**

Crimean-Congo hemorrhagic fever virus (CCHFV) and Rift Valley fever virus (RVFV) are emerging arboviruses primarily affecting domestic animals. Research has demonstrated their endemicity in various parts of the world, including Africa. Despite the high seroprevalence of CCHFV infection recently reported among cattle in Cameroon, the epidemiological status of animal workers who frequently interact with these animals remains poorly understood. This study investigates the seroprevalence of CCHFV and RVFV infections, along with associated factors, among animal workers in Cameroon.

**Methods:**

Between May 2023 and January 2024, blood samples were collected from shepherds, slaughterers, butchers, and veterinarians at farms, slaughterhouses, and livestock markets in Centre and North regions in Cameroon. Serum samples were analyzed for Immunoglobulin G (IgG) antibodies against CCHFV and RVFV using the enzyme-linked immunosorbent assay (ELISA) and neutralization tests. Univariate and multivariable analyses were conducted using R statistical software version 4.1.

**Results:**

Seroprevalence estimates of CCHFV and RVFV among high-risk population were 4.6% (36/790) and 9.9% (78/790), respectively. Multivariable analysis revealed that participants with 21–40 years or more than 41 years of professional experience were significantly associated with higher RVFV seroprevalence [aOR = 2.31; 95% CI: 1.02–5.23 and aOR = 5.34; 95% CI: 1.84–15.58, respectively] compared to those with 1–5 years of experience, suggesting that RVFV seroprevalence increases with longer employment duration. None of the variables analyzed were associated with CCHFV occurrence.

**Discussion:**

Our study confirms the presence of CCHFV and RVFV antibodies among animal workers. These viruses are likely endemic in Cameroon, despite the absence of reported clinical cases. We recommend implementing regular surveillance and preventive measures to protect at-risk populations.

## Introduction

Crimean-Congo hemorrhagic fever (CCHF) is the most widespread tick-borne viral disease in humans. The causative agent, CCHFV, was first isolated in 1944–1945 in Crimea during an epidemic among soviet soldiers (1). It can be transmitted to animals and humans mainly through the bite of ticks belonging to the genus *Hyalomma*, but can also be transmitted by direct contact with the blood, secretions, organs or other bodily fluids of infected people or animals (2). CCHFV can infect many species, however, domestic animals such as cattle, sheep, goats, are the most frequently infected, developing viremia without any particular clinical signs or symptoms (3). In humans, the disease begins with flu-like symptoms, headaches, high fever, vomiting, myalgia and sometimes blindness, which, depending on the severity of the infection, can progress to severe hemorrhaging and death if left untreated. The current case-fatality rate ranges from 10 to 40% (4). CCHF is endemic in Africa, Asia, Middle East and Eastern Europe (5). In some countries that are not endemic to CCHF, traces of exposure to CCHFV have been found, demonstrating the rapid spread of the virus into new territories and the consequent increase in the number of endemic countries in the coming years (6).

Rift Valley fever (RVF), also known as enzootic sheep hepatitis, is a viral zoonosis that affects both animals (ruminants) and humans, given their cohabitation (7). It was discovered in 1912 during an epizootic that caused numerous deaths in sheep, and in 1930, the virus responsible for the disease, RVFV, was isolated in Kenya (8). After Kenya, RVFV was identified in sub-Saharan Africa and over time became endemic in many African countries and the Arabian Peninsula (9). It is transmitted to humans and animals through mosquito bites or contact with infected tissues and fluids (33). The disease causes mortality in newborn lambs and abortions in pregnant females (10). In humans, RVF is approximately 98% asymptomatic, with only 2% of those infected showing mild fever, myalgia and among more serious cases, blindness, hemorrhagic fever, meningoencephalitis and hepatitis which can lead to death (34). RVFV epidemics have been documented in several parts of the world, including Kenya, Egypt, Somalia and Rwanda, with prevalence and mortality rates varying according to species (11). These epidemics are more often linked to climatic events that improve mosquito breeding conditions, leading to a significant increase in infection rates, with serious consequences for public health (12). The mortality rate is dependent on the epidemic (9). In addition, economic losses are considerable, particularly in regions that rely on agriculture and the livestock trade (13). A systematic review has shown that the human prevalence of RVFV is 7.8%, with a mortality of 27.5% in Africa (14).

Crimean-Congo Hemorrhagic Fever Virus (CCHFV) and Rift Valley fever virus (RVFV) belong to the Bunyavirales order, although their families and genera differ. CCHFV belongs to the *Nairoviridae* family and the genus *Orthonairovirus*, while, RVFV is a member of the *Phenuiviridae* family and the genus *Phlebovirus* (15, 16). These two arboviruses are on the list of 10 emerging infectious diseases for which the World Health Organization (WHO) recommends that utmost importance be given to monitoring (35).

In Cameroon, serological tests have been carried out much more frequently on cattle, sheep, goats, etc. indicating a seroprevalence of CCHFV of up to 98% in cattle (17, 18). However, few studies have been carried out in humans on RVFV and the first by Paix et al. (19), showed an RVFV seroprevalence of 1.1%. A year later, in 1989, Gonzalez et al. (20) conducted a study in various African regions, including Cameroon, to detect antibodies to RVFV and CCHFV. This study revealed a seroprevalence of 0.2 and 0.6%, respectively, for CCHFV and RVFV. Furthermore, in the eastern region of Cameroon in 2018, Sadeuh and colleagues reported seroprevalences of CCHFV (4.4%) and RVFV (12.4%) (21). These studies showed a significant increase in RVFV and CCHFV seroprevalence rates in humans over time. Despite this observation, there is a lack of systematic epidemiological studies assessing the seroprevalence of these infections in the general population. This is particularly true for workers in the animal sector, such as slaughterers, butchers, breeders and veterinarians, who are in close contact with livestock. They are at risk of infection through exposure to tick bites and body fluids from viremic animals (22). The objectives of this study were to determine the seroprevalence of RVFV and CCHFV in people living in close contact with livestock and identify associated factors, thereby providing essential data for improving infection prevention and management in these populations.

## Materials and methods

### Ethical consideration

This study was approved by the National Ethics Committee for Research on Human Health (CNERSH) (N° 2021/10/1394/CE/CNERSH/SP) and the Ministry of Public Health (Administrative authorization N° 631-03.22). Written informed consent was obtained from all study participants before blood collection.

### Study design, area and population

**Figure 1:**
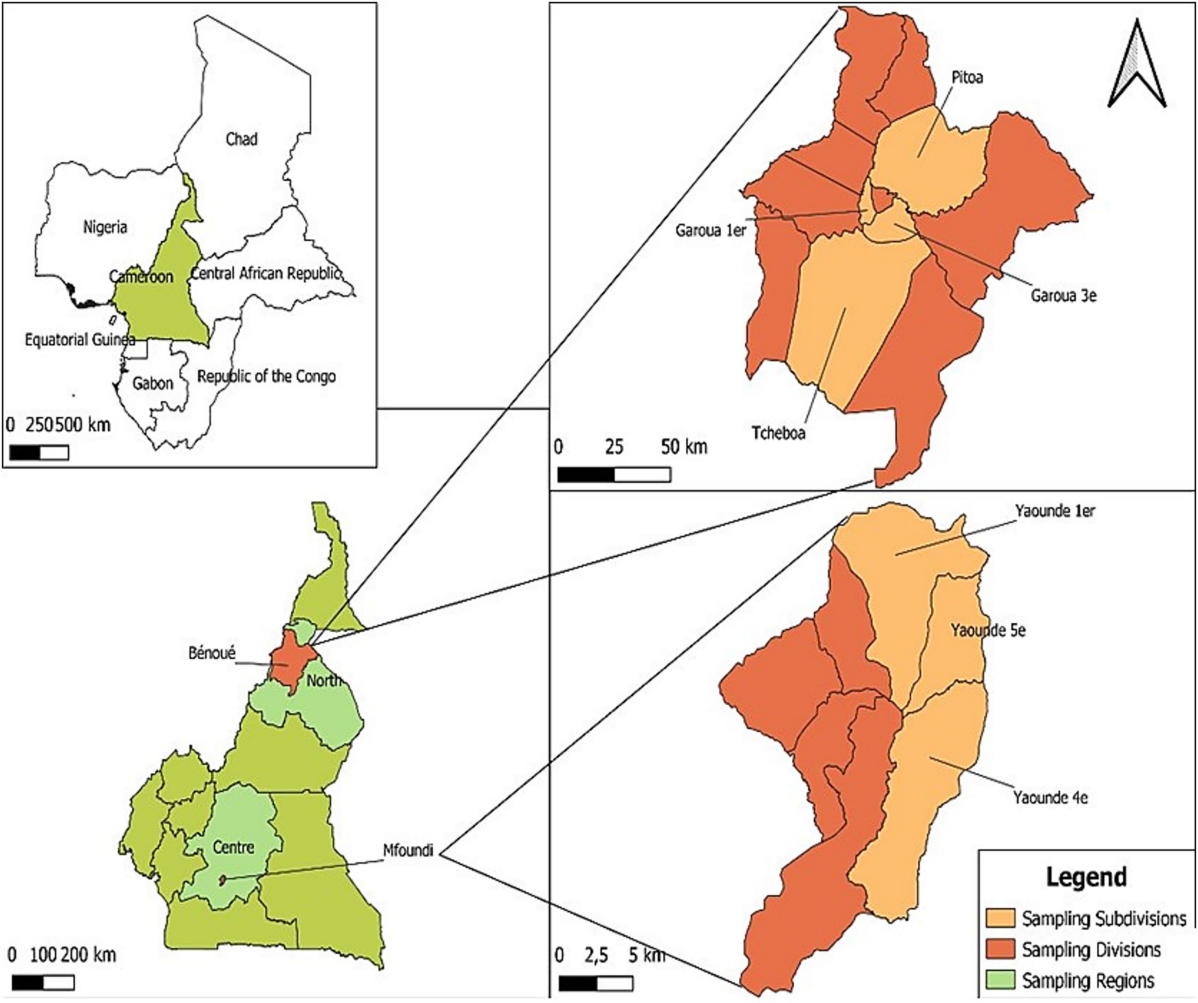
Map of the study area. This map shows the different regions of Centre and North Cameroon where human samples were collected. The regions are shown in turquoise green, while the cities of Yaoundé and Garoua are shown in coral. The districts sampled are shown in yellow. The map was created using QGIS 3.14.16 and open access shared files.

A cross-sectional study was conducted between May 2023 and January 2024 in the Centre and North regions of Cameroon, specifically in Garoua and Yaoundé (Figure 1). Garoua, located at approximately 9.31° N and 13.39° E, experiences a Soudano-sahelian climate with an average temperature of 27 °C, which can rise to 40 °C. Annual rainfall ranges from 800 to 1,200 mm, with a rainy season from July to October, while the remaining months are dry. Livestock farming in this area is primarily extensive, relying on nomadic herding and natural pastures. The main livestock includes cattle, sheep, goats and poultry. The population in Garoua consists mainly of shepherds whose livelihoods are closely intertwined with their domestic animals. Yaoundé, located at approximately 3.87° N and 11.50° E, has an equatorial climate with an average annual temperature of 24 °C and rainfall between 1,500 and 2,000 mm. The rainy season lasts from March to November. As a central hub for livestock in Cameroon, Yaoundé receives animals from various regions of the country.

Samples were collected from individuals in five slaughterhouses, four livestock markets and four farms. Participants were selected using convenience sampling method. Selection criteria for slaughterhouses included species specialization, daily slaughter volume, and geographic location. For livestock markets, factors such as seller availability and accessibility were considered. Farms were chosen based on accessibility, the presence of at least 50 animals and their interactions with local markets.

### Samples collection in humans in close contact with cattle, sheep and goat

Apparently healthy individuals (animal workers) in close contact with livestock (shepherds, butchers, slaughterers and veterinarians) were included in the study after providing signed informed consent. Information on participant demographics (age, sex), occupation (type, duration), exposure to ticks/mosquitoes, raw milk and meat consumption were collected using a standardized questionnaire. Subsequently, blood was collected from participants via venipuncture in dry tubes using standard medical phlebotomy procedures. To ensure participant protection and the quality of collected samples, we followed the WHO recommendations regarding blood sampling in individuals (23). Briefly, we began by informing the participant about the purpose of the procedure, the steps to follow, and any potential adverse effects. Next, we gathered the necessary materials, including sterile gloves, appropriate collection tubes, puncture equipment (needle and collection device), alcohol gauze, and bandages. We then performed handwashing before and after the procedure, wearing sterile gloves to prevent contamination. We identified the appropriate vein, typically the cephalic or basilic vein in the antecubital fossa, while avoiding areas with infections, scars, or varicose veins. After disinfecting the puncture site with an alcohol swab, we applied a tourniquet to dilate the vein and inserted the needle at an angle of 15–30° toward the vein. Once the blood was collected, we removed the needle and applied pressure to the puncture site using gauze. We labeled the tubes immediately after collection and placed them in a refrigerated unit. We monitored the patient for any potential side effects and provided instructions on post-collection care, including the prohibition of physical activities. Finally, we recorded the details of the collection, such as the date, time, type of sample, and participant code.

After collection, blood specimens were transferred to the laboratory in a refrigerated safety unit for processing. Samples were centrifuged at 2500 rpm for 10 min in the laboratory, and the resulting serum were aliquoted and stored at −80 °C until use.

### Enzyme-linked immunosorbent assay (ELISA)

To detect anti-CCHFV and anti-RVFV antibodies, samples were analyzed using a modified capture sandwich ELISA protocol from the Pasteur Institute of Dakar (IPD) (24). This in-house technique uses ascites and antigens (both positive and controls) produced by the WHO Collaborating Centre for Arboviruses and Haemorrhagic Fever Viruses, located at IPD.

Briefly, 96-well microplates were coated with CCHFV or RVFV immune ascites produced in mice and diluted 1,000-fold in carbonate–bicarbonate buffer and incubated overnight at 4 °C. The following day, 200 μL of blocking buffer (1X phosphate-buffered saline, 0.05% tween 20, 1% skimmed milk and 0.1% fetal bovine serum) was added to each well and the microplates were incubated at room temperature for 30 min. Subsequently, 100 μL of control and positive antigens, diluted 100-fold in dilution buffer (1X phosphate-buffered saline, 0.05% tween 20 and 2% skimmed milk) were added the corresponding wells (one well for each antigen per sample) and the microplate was incubated at 37 °C for 1 h. Unbound antigens were washed away, and 100 μL of samples, including positive and negative controls diluted 100-fold in dilution buffer, were added to corresponding wells, followed by incubation at 37 °C for 1 h. Next, the secondary antibody, goat anti-human IgG antibodies conjugated with Peroxidase-Labeled (Sigma-Aldrich) diluted at 1:10000 in dilution buffer was added to all wells and the plate was incubated at 37 °C for 1 h. After each incubation, the plates were washed five times with 350 μL wash buffer (1X phosphate-buffered saline + 0.05% tween 20) using an automatic plate washer (Wellwash, Thermoscientific). Finally, 100 μL of 3,3′,5,5′-tetramethylbenzidine (TMB) substrate was added to each well and incubated for 5 min at room temperature. The reaction was stopped by adding 100 μL of 4 N H_2_SO_4_. Optical densities (OD) were read at 450 nm (with 620 nm as reference filter) with an automated microplate reader (MultiSkan FC, Thermoscientific).

The ΔOD (OD of positive antigen-OD of negative antigen) was determined for each sample and controls. Samples with ΔOD ≥ 0.2 were classified as positive, while samples with ΔOD < 0.2 were classified as negative for the corresponding arbovirus (Supplementary Figure 1).

### Neutralization tests for CCHV and RVFV

HEK293T and Vero E6 cells were cultured in Dulbecco’s Modified Eagle’s Medium (DMEM) (Sigma-Aldrich, St. Louis, MO, USA) supplemented with 10% fetal bovine serum (FBS) (Sigma-Aldrich, St. Louis, MO, USA) and penicillin–streptomycin (0.1 mg/mL). These cells were maintained at 37 °C in a CO_2_ incubator.

For CCHFV, Vesicular Stomatitis Indiana Virus (VSIV) pseudotyped with CCHFV-GP (VSVΔG*CCHFV-GPc) was generated as described previously with slight modifications (25, 26). Briefly, HEK293T cells were transfected with a mammalian expression plasmid encoding the CCHFV glycoprotein precursor (pCAGGS-Gp) using TransIT®-LT1 transfection reagent (Mirus Bio LLC, WI, USA) according to the manufacturer’s instructions. Thirty-six hours after transfection, the VSVΔG*-G containing the green fluorescent protein (GFP) gene instead of the receptor-binding VSV-G protein gene was used to infect transfected cells at a multiplicity of infection of ≥1 for 1 h at 37 °C, with gentle rocking every 15 min. The inoculum was then removed and the plates were carefully washed twice with 8 mL of DMEM lacking FBS. Next, 4 mL of DMEM containing 10% FBS was added. The culture supernatant was harvested 12–14 h after incubation at 37 °C in a CO₂ incubator. The supernatant containing VSVΔG*CCHFV-GPc was centrifuged at 2,000 *g* for 15 min at 4 °C and stored at −80 °C until use. The number of infectious units (IUs) contained in the stock virus was determined using Vero E6 cells as described elsewhere (26). Before neutralization tests, the virus was pre-treated with a neutralizing monoclonal antibody to VSIV G protein (VSV-G[N]1–9) at room temperature for 20 min to abolish the residual parent VSVΔG*-G (27). For the neutralization test, VSVΔG*CCHFV-GPc was diluted to obtain 500–1,500 infectious units and mixed with an equal volume of diluted human serum (1:100). Next, the virus–serum mixture was incubated at room temperature for 30 min and inoculated into confluent Vero E6 cells grown in 96-well plates (1.5 × 10^4^ cells seeded per well 20 h earlier). Infected cells were counted 20 h post-inoculation using an IN-Cell Analyzer 2500HS (GE Healthcare, Waukesha, WI, USA). The relative percentage of infectivity was determined by setting the number of cells infected without serum to 100%. A sample was considered positive if the relative percentage of infectivity was less than 50% (Supplementary Figure 2).

The neutralization test for RVFV was conducted using Vero E6 cells. For the experiment, 1.0 × 10^5^ cells per well were seeded into 24-well plates and incubated overnight at 37 °C in a CO₂ incubator. On the following day, sera were diluted (1:10), followed by two-fold serial dilutions up to 1:80, in DMEM containing 2% FBS and 1% penicillin–streptomycin. Subsequently, 150 μL of diluted virus [80 plaque-forming unit (PFU)/125 μL] was added to an equal volume (150 μL) of the diluted serum mixture, which was then thoroughly mixed by suction-breathing and incubated for 1 h at room temperature. Human serum, commercially available and purchased from Thermo Fisher Scientific, was prepared as negative control. The virus-serum mixture (250 μL) was inoculated on to Vero E6 cells and incubated for 1 h at 37 °C with a consistent supply of CO_2_. Following this step, the mixture was removed and the plates were washed with PBS before adding 500 μL of Minimum Essential Medium containing 2% FBS, 1% penicillin–streptomycin, and 1% methylcellulose. The plates were then incubated at 37 °C in a CO_2_ incubator. Five days post-incubation, the supernatant was discarded, and each well was fixed with 10% formaldehyde neutral buffer solution containing 0.4% crystal violet at 4 °C overnight. The following day, excess formaldehyde and crystal violet were removed, plates were washed with water, then covered with blotting paper to dry them thoroughly, after which the number of plaques were counted. The plaque reduction percentage was calculated as (PFU in sample well/PFU in control well) × 100. Samples with >50% plaque reduction were considered positive.

### Data analysis

Data obtained were analyzed using R Software Version 4.1 (28). Descriptive analyses were then performed to compare demographic and exposure-related variables (location, age group, sex, raw meat or milk consumption, employment, employment duration, Sting-ticks) across serostatus categories. Categorical variables were summarized as frequencies and percentages, and compared using Chi-square or Fisher’s exact tests.

To identify independent risk factors associated with arboviral seropositivity, univariate logistic regression analyses were first performed for each potential explanatory variable. All variables with a *p-value* <0.25 in univariate analysis were considered eligible for inclusion in a multivariable logistic regression model. The final model was selected using the Akaike Information Criterion (AIC) to retain the most parsimonious set of predictors. Adjusted odds ratios (aOR) with 95% confidence intervals (95% CI) were reported for the final models. Statistical significance was defined as *p* < 0.05.

## Results

A total of 790 blood samples were collected from individuals in regular contact with livestock, including 135 slaughterers, 108 shepherds, 422 butchers, and 125 veterinarians. Samples were collected in slaughterhouses, farms and livestock markets located in four districts of the Centre Region (Yaoundé 1, Yaoundé 4, Yaoundé 5 and Yaoundé 6) and four districts of the North Region (Garoua I, Garoua III, Tcheboa and Pitoa).

### Seroprevalence and univariate analysis of CCHFV in humans

Among 790 sera collected from animal workers, 36 (4.56%) were positive for anti-CCHFV IgG. From univariate analysis, no significant associations were observed between region, sex, raw milk consumption, employment duration, job type, frequency of exposure to livestock and CCHFV seroprevalence (Table 1). Although not statistically significant, the CCHFV seroprevalence in the North region (5.2%) was higher than in the Centre region (3.7%). Similarly, participants aged 45 and over seemed to have higher seroprevalence than those under 45.

**Table 1:** Sociodemographic characteristics, seroprevalence and univariate analysis for CCHFV in humans.

Variable	Total (*N* = 790)	Number of positive (*N* = 36)	seroprevalence (4.6%)	Univariate analysis		*p*
				OR	CI	
Regions						
Centre	324	12	3.7		–	
North	466	24	5.2	1.41	(0.71–2.96)	0.340
Sex						
Female	78	4	5.1		–	
Male	712	32	4.5	0.87	(0.33–2.98)	0.799
Age						
[10–14]	12	0	0.0			
[15–24]	144	7	4.8			
[25–44]	457	19	4.2			
[45–64]	154	9	5.8			
≥65	16	1	6.3			**0.836**
Raw_milk_consumption						
No	429	14	3.3		–	
Yes	357	22	6.2	1.95	(0.99–3.95)	0.057
Raw_meat_consumption						
No	667	27	4.0			
Yes	121	9	7.4			
Employment						**0.100**
Slaughterers	135	3	2.2		–	
Shepherd	108	10	8.4	4.04	(1.17–18.57)	0.040
Butcher	422	19	4.5	2.07	(0.69–8.93)	0.246
Veterinarians	125	5	4.0	1.83	(0.44–9.09)	0.413
Employment duration						
[1–5]	177	7	4.0		–	
[6–10]	175	9	5.1	1.32	(0.48–3.76)	0.594
[11–20]	221	12	5.4	1.39	(0.55–3.82)	0.495
[21–40]	173	4	2.3	0.57	(0.15–1.94)	0.384
≥41	30	3	10.0	2.70	(0.56–10.37)	0.168
Exposure frequency						
Weekly	34	2	5.8		–	
Never	16	1	6.3	1.07	(0.05–12.00)	0.959
Occasionally	56	1	1.8	0.29	(0.01–3.15)	0.321
Daily	678	32	4.7	0.79	(0.23–5.02)	0.757
Sting-ticks						
No	314	9	2.9			
Yes	459	27	5.9			**0.051**
Total	**790**	**36**	**4.6%**			

CCHFV, Crimean-Congo Hemorrhagic Fever Virus; *p*, *p*-value; *N*, Total; OR, Odds Ratio; CI, Confidence Interval.

### Seroprevalence and univariate analysis of RVFV in humans

The analysis revealed an overall seroprevalence of RVFV in humans of 9.87% (78/790), with a higher prevalence in the North Region (13.3%) compared to the Centre Region (4.9%) (Table 2). Significant variables included the region, where individuals sampled in the North were significantly more likely to be seropositive (OR = 2.95; 95% CI: 1.71–5.39). Additionally, participants consuming raw cow’s milk had an increased risk of seropositivity (OR = 2.19; 95% CI: 1.36–3.59). Employment duration also influenced seropositivity, with participants having more than 40 years of employment showing higher odds (OR = 8.00; 95% CI: 2.84–22.79), while those with 6–10 years of employment had lower odds (OR = 1.25; 95% CI: 0.51–3.18). Regarding occupational groups, shepherds had the highest seroprevalence (14%). Conversely, no significant differences were observed by sex (10.3% in males and 6.4% in females), nor based on mosquito bites, employment, or frequency of exposure to livestock (Table 2).

**Table 2:** Sociodemographic characteristics, seroprevalence and univariate analysis for RVFV in humans.

Variable	Total (*N* = 790)	Number of positive (*N* = 78)	seroprevalence (9.9%)	Univariate analysis		*p*
				OR	CI	
Regions						
Centre	324	16	4.9		–	
North	466	62	13.3	2.95	(1.71–5.39)	<0.001
Sex						
Female	78	5	6.4		–	
Male	712	73	10.3	1.67	(0.72–4.87)	0.285
Age						
[10–14]	12	1	8.3			
[15–24]	144	6	4.2			
[25–44]	457	42	9.2			
[45–64]	154	23	14.9			
≥65	16	6	37.5			<0.001
Raw_milk_consumption						
No	429	29	6.8		–	
Yes	357	49	13.7	2.19	(1.36–3.59)	<0.001
Raw_meat_consumption						
No	667	62	9.3			
Yes	121	16	13.2			
Employment						
Slaughterers	135	7	5.2		-	
Shepherd	108	16	14.0	2.98	(1.21–8.08)	<0.022
Butcher	422	50	11.8	2.46	(1.16–6.06)	0.031
Veterinarians	125	6	4.8	0.92	(0.29–2.85)	<0.887
Employment duration						
[1–5]	177	9	5.0		–	
[6–10]	175	11	6.3	1.25	(0.51–3.18)	0.627
[11–20]	221	23	10.4	2.17	(1.01–5.06)	0.057
[21–40]	173	25	14.5	3.15	(1.48–7.34)	0.005
≥41	30	9	30.0	8.00	(2.84–22.79)	<0.001
Exposure frequency						
Weekly	34	3	8.8		–	
Never	16	0	0.0	00	0.00–24.71	
Occasionally	56	2	3.6	0.38	(0.05–2.43)	
Daily	678	73	10.8	1.25	(0.43–5.28)	0.182
Sting-mosquitoes						
No	314	28	8.9			
Yes	459	48	10.5			0.480
Total	790	78	9.9%			

RVFV, Rift Valley fever virus; *p*, *p*-value; *N*, Total; OR, Odds Ratio; CI, Confidence Interval.

### Multivariable analysis

In multivariable analysis, employment duration was significantly associated with RVFV seroprevalence. Compared to participants with 1–5 years of professional experience (reference group), those with 21–40 years (aOR = 2.31; 95% CI: 1.02–5.23; *p* = 0.045) and more than 41 years of experience (aOR = 5.34; 95% CI: 1.84–15.58; *p* = 0.002) were significantly more likely to be seropositive R (Table 3).

**Table 3:** Factors associated with RVFV exposure in humans.

Employment duration	Multivariable analysis		
	OR	CI	*p*
[1–5]		–	
[6–10]	1.25	(0.50–3.13)	0.625
[11–20]	1.79	(0.79–4.04)	0.160
[21–40]	2.31	(1.02–5.23)	0.045
[41]	5.34	(1.84–15.58)	0.002

RVFV, Rift Valley fever virus; *p*, *p*-value; OR, Odds Ratio; CI, Confidence Interval.

### RVFV and CCHFV neutralization results

Following the analysis of human sera via ELISA for anti-CCHFV and anti-RVFV IgG antibodies, a total of 100 samples were selected for the neutralization tests. Notably, none of these samples had antibodies neutralizing VSVΔG*CCHFV-GPc, although 33/100 samples tested positive for anti-CCHFV IgG ELISA. In contrast, antibodies neutralizing RVFV were detected in 44/100 samples. Interestingly, seven samples positive on ELISA were negative for neutralizing antibodies, and seven other samples that tested positive for neutralizing antibodies were negative on ELISA. Overall, 37 ELISA-positive samples had antibodies neutralizing RVFV (Table 4).

**Table 4:** CCHFV and RVFV neutralization results.

Virus	Total	ELISA +	Neutralisation +	Positive N, E	Negative N, E	Positive N, −E	Negative N, +E
CCHFV	100	33	0	0	67	0	33
RVFV	100	44	44	37	49	7	7

N, Neutralization; E, ELISA; “−”, Negative; “+”, Positive.

## Discussion

This study aimed to assess the seroprevalence of CCHFV and RVFV among individuals at high risk in the Centre and North regions of Cameroon.

Only few studies have been carried out on the seroprevalence of CCHFV and RVFV in humans, with findings indicating low seroprevalence estimates. For the first time in Cameroon, this study highlighted the exposure of high-risk individuals to CCHFV and RVFV, with a seroprevalence of 4.6 and 9.9%, respectively. The observed seroprevalence of anti-CCHFV and anti-RVFV antibodies indicates previous exposure of animal workers to these viruses. In Cameroon, CCHFV- and RVFV-specific antibodies have never been tested in slaughterers, shepherds, butchers, or veterinarians. Nevertheless, a study conducted among Pygmy populations in the East Region of Cameroon in 2018 by Sadeuh-Mba et al. (21) reported a CCHFV seropositivity of 4.4%, which is comparable to our finding (4.6%), despite differences in participant profiles. The same study recorded a RVFV seroprevalence of 12.4%. Diagnostic methods, sampling locations, target populations, seasons of sample collection and local climatic conditions may influence results. For example, the dual-antigen ELISA used in most studies detects both CCHFV IgG and IgM (29) and uses a larger volume of serum samples (30), which may contribute to higher seroprevalence, unlike other types of ELISA that detect only IgG or IgM, such as the one used in this study.

Anti-CCHFV IgG antibodies were detected in animal workers across both regions, with a higher seroprevalence found among shepherds (breeders) at 8.4%. This may be attributed to their daily interaction with domestic animals, increasing their exposure to tick bites and potentially viremic fluids. In our study, the seropositivity rate for CCHFV progressively increased with age, as evidenced by the higher seroprevalence (6.3%) observed in the older age group (≥ 65 years). This suggests that older participants may have been more exposed to CCHFV throughout their lives, resulting in higher seropositivity.

Anti-RVFV IgG antibodies were detected in participants from both regions, with the highest positivity rate observed in the Northern region at 13.3%. This high seroprevalence may be attributed to the fact that livestock rearing is a primary activity in this region, exposing nearly the entire population to livestock and consequently increasing the risk of RVFV exposure. Participants with 21–40 years of professional experience and those with over 41 years of experience were, respectively, at least twice and five times more likely to be seropositive compared to participants with 1–5 years of experience. This trend indicates that RVFV seroprevalence may increase with longer employment duration, suggesting a potential cumulative occupational exposure over time.

Of the previous seroprevalence studies conducted in Cameroon, none has assessed neutralizing antibodies targeting the CCHFV and RVFV in individuals at high risk of exposure to livestock. Our study detected neutralizing antibodies to RVFV in the analyzed sera, consistent with results obtained by ELISA. However, seven ELISA-negative sera turned out to be positive in the serum neutralization test. These results may indicate either ELISA false negatives or the presence of substances others than antibodies that inhibit-ELISA without affecting serum neutralization. It is also possible that these sera contain compounds capable of inhibiting viral infections during seroneutralization and could be attributed to poor serum sample quality. No neutralizing antibodies to CCHFV were detected in any of the 100 samples analyzed. This could be explained either by low levels of neutralizing antibodies to CCHFV or by late sampling, i.e., 5 months or more after exposure. Previous studies have shown that individuals infected with CCHFV produce only low levels of neutralizing antibodies, which are typically highest during the second and third weeks after infection before dropping considerably and becoming undetectable 19 weeks after exposure (31). This result is consistent with finding reported by Bohou Kombila et al. (32), where no positive ELISA result was confirmed by seroneutralization. The discrepancy results obtained with CCHFV seroneutralization might raise questions about the sensitivity and specificity of the ELISA used in this study. However, it is important to note that these two tests used in this work, target different types of antibodies. The CCHFV ELISA test detects IgG antibodies directed against the nucleoprotein, whereas the seroneutralization test targets neutralizing antibodies specific to the glycoprotein via its neutralizing epitopes.

Based on the CCHFV and RVFV seroprevalence results presented in this study, in the absence of reported epidemics, it would be appropriate to focus research efforts on these pathogens in Cameroon. Indeed, CCHF and RVF are known for their potential sudden emergence in humans. To avoid any unexpected epidemics, it is essential to monitor these diseases, even if no clinical cases have yet been identified in Cameroon. This study has some limitations resulting from a lack of information; on the use of personal protective equipment when handling animals, the management of dead animals, the vaccination of live cattle and the disinfection of pens or farms with acaricides. Furthermore, research conducted on low-risk individuals who have never been in contact with animals would have helped determine whether the presence of antibodies in high-risk individuals was a direct result of their exposure to livestock.

## Conclusion

This study highlights exposure to CCHFV and RVFV among shepherds, slaughterers, butchers and veterinarians, suggesting a long-standing circulation of these pathogens in these populations in Cameroon. The seropositivity observed is linked to cumulative exposure associated with age and employment duration, indicating that circulation of these viruses may extend beyond specific high-risk populations. There is need for tailored interventions and a better understanding of local transmission dynamics. In addition, further research is required to characterize virus strains circulating in Cameroon and to study the broader transmission patterns to develop more effective disease management strategies.

## Data Availability

The original contributions presented in the study are included in the article/Supplementary material, further inquiries can be directed to the corresponding author.
