# Supplementary material for: Detection of Crimean-Congo hemorrhagic fever virus and Rift Valley fever virus antibodies in animal workers in Cameroon
Source: Front Vet Sci. 2025 Nov 28;12:1646715. doi: 10.3389/fvets.2025.1646715 (PMC12699157; doi:10.3389/fvets.2025.1646715)
Supplement: Supplementary file 1 [file Data_Sheet_1.PDF]

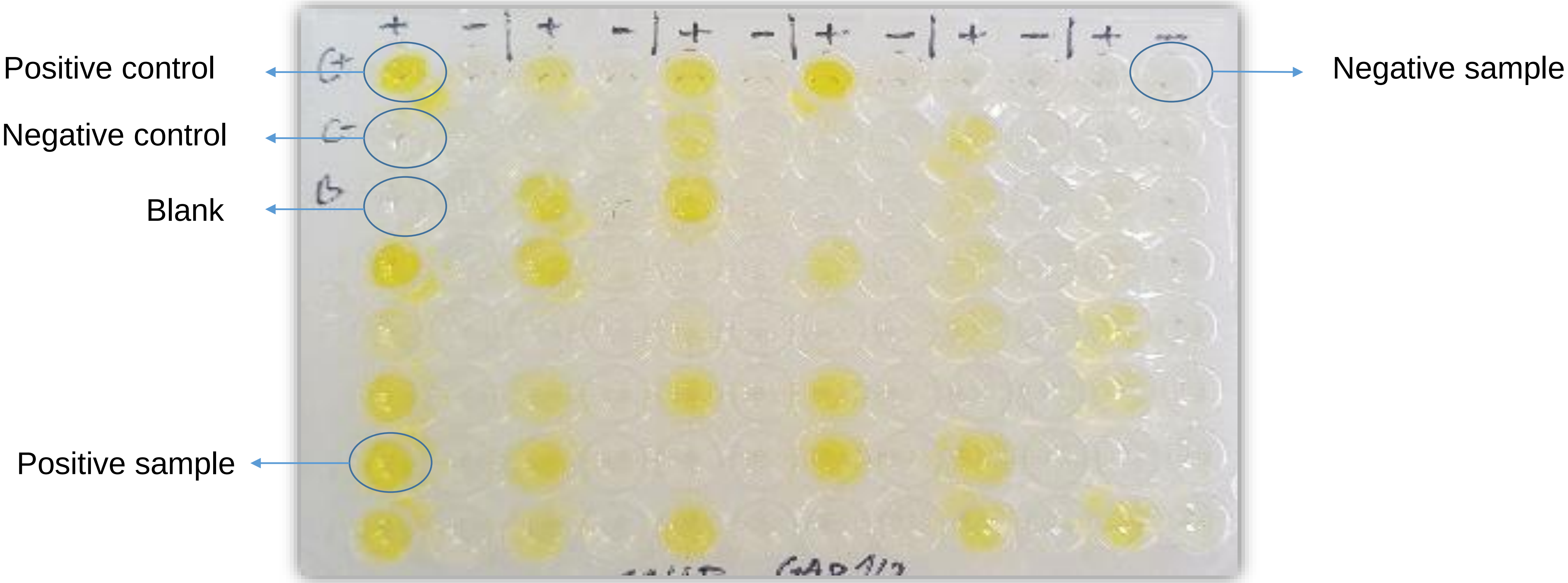

Supplementary figure 1: This Figure illustrates the results of a plate obtained following the ELISA procedure. The first well contains the positive control (C+), the second well the negative control (C-), and the third well is the blank. The wells colored in yellow indicate positive samples, while the uncolored wells correspond to negative samples. **“+”** : positive antigen and **“-”** : Control antigen,

A. CCHFV neutralization **B. RVFV neutralization**

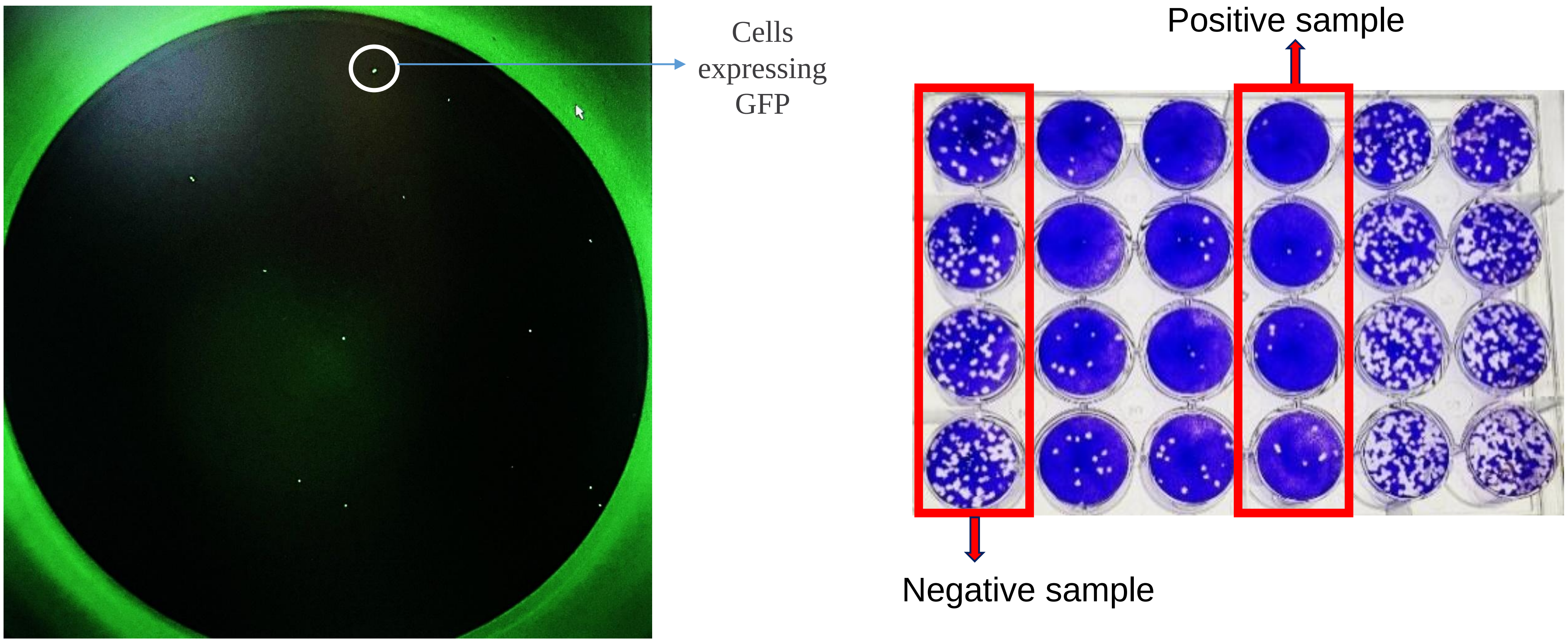

Supplementary figure 2 : Results of the neutralization tests : CCHFV (A) and RVFV (B)
